# Adaptive Segmentation of Remote Sensing Images Based on Global Spatial Information

**DOI:** 10.3390/s19102385

**Published:** 2019-05-24

**Authors:** Muqing Li, Luping Xu, Shan Gao, Na Xu, Bo Yan

**Affiliations:** 1 School of Aerospace Science and Technology, XIDIAN University, 266 Xinglong Section of Xifeng Road, Xian 710126, China; mqli126@stu.xidian.edu.cn (M.L.); boyan@xidian.edu.cn (B.Y.); 2 Research Institute of Vibration Engineering, ZhengZhou University, 100 Kexue Avenue of Gaoxin Section, ZhengZhou 450001, China; shanggao@gs.zzu.edu.cn; 3 School of Life Sciences and Technology, XIDIAN University, 266 Xinglong Section of Xifeng Road, Xian 710126, China; nxu_1994@stu.xidian.edu.cn

**Keywords:** image segmentation, global spatial information, adaptive parameters, strong denoising

## Abstract

The problem of image segmentation can be reduced to the clustering of pixels in the intensity space. The traditional fuzzy c-means algorithm only uses pixel membership information and does not make full use of spatial information around the pixel, so it is not ideal for noise reduction. Therefore, this paper proposes a clustering algorithm based on spatial information to improve the anti-noise and accuracy of image segmentation. Firstly, the image is roughly clustered using the improved Lévy grey wolf optimization algorithm (LGWO) to obtain the initial clustering center. Secondly, the neighborhood and non-neighborhood information around the pixel is added into the target function as spatial information, the weight between the pixel information and non-neighborhood spatial information is adjusted by information entropy, and the traditional Euclidean distance is replaced by the improved distance measure. Finally, the objective function is optimized by the gradient descent method to segment the image correctly.

## 1. Introduction

In recent years, clustering technology has played an important role in remote sensing image segmentation. The technique uses visual features such as image color, texture and shape to gather together areas with large similarity, so that the pixels in the same area are as similar as possible, and the pixels in different areas are as different as possible [1,2,3,4]. The fuzzy c-means (*FCM*) algorithm has the advantages of conforming to human cognitive characteristics, easy implementation, simple description and good segmentation effect [5]. Due to the *FCM* algorithm using fuzzy membership to measure the degree of pixels belonging to a certain class relative to the other segmentation algorithms, it can retain the original image information as much as possible [6]. It has been widely used in medicine and remote sensing image segmentation [7,8,9,10]. But the traditional *FCM* algorithm fails to consider the correlation between the grey features of each point and its neighborhood pixels in image segmentation, which makes the algorithm more sensitive to noise, low contrast, intensity inconsistency, and so on [11]. When imaging remote sensing images, due to the constraints of satellite imaging technology, problems such as unclear pixels, discrete pixels or block-forming pixels appear, which seriously affect the segmentation effect of *FCM* algorithm. In order to effectively solve these problems, researchers have proposed many improved *FCM* algorithms. Ahmed et al. [12] added spatial neighborhood information around pixels to the *FCM* algorithm, proposing the *FCM*_S algorithm. The objective function is modified to increase the robustness of the algorithm to noise points and improve the precision of the segmentation results. However, the *FCM*_S algorithm takes a long time to calculate the relationship between each pixel point and the surrounding neighborhood, resulting in high computational complexity and low efficiency. So, Chen and Zhang [13] proposed improved algorithms, *FCM*_S1 and *FCM*_S2. Before iterative calculation, these algorithms first evaluate the influence of neighborhood pixels on the center pixel, which is equivalent to filtering the image. The algorithm avoids repeated computation in iterations and reduces the time complexity of the algorithm effectively. Mean filtering and median filtering are used respectively in *FCM*_S1 and *FCM*_S2, which have good segmentation effect for images with Gauss noise and salt and pepper noise. Cai et al. [14] introduced a new local similarity measurement method, combined with local spatial distance and grey difference, and proposed the fast generalized fuzzy -means (FG*FCM*) clustering algorithms. This algorithm not only considers the spatial information of neighborhood pixels in the filtering function, but also considers the grey information of neighborhood pixels, which can better preserve the details of the image while filtering. In the above improved *FCM* algorithm, there are parameter settings, which have a significant impact on the segmentation results. Krinidis et al. [15] defined a new fuzzy factor, combining local spatial information and grey level information, and proposed the fuzzy local information C-Means (*FLICM*) algorithm. The algorithm effectively integrates the spatial information and grey level information of the neighborhood pixels, enhances the insensitivity of the algorithm to noise, and controls the weight between denoising and image details through adaptive adjustment of parameters. When the image noise is relatively serious, the neighborhood information of the pixel may also be polluted, so the neighborhood information based on the local space of the image cannot play an active guiding role in the image segmentation, making the fuzzy clustering algorithm that integrates the local space information unable to meet the requirements of high-precision image segmentation. To solve this problem, Zhao et al. [16] proposed a fuzzy c-means clustering algorithm based on non-local spatial information (the *FCM*_NLS algorithm). The algorithm first uses the non-local spatial information of image pixels to filter the original image, and then directly calls the result in the iteration, narrowing the time complexity of the algorithm. However, the *FCM*_NLS algorithm ignores the non-uniformity of noise distribution, so it is sensitive to noise and still has yet to be improved. Gong et al. [17] proposed a fuzzy c-means clustering algorithm based on local information and kernel metric (the KW*FLICM* algorithm). On the basis of the *FLICM* algorithm, this algorithm introduces kernel space and a similarity measurement factor, which greatly improve the segmentation effect and denoising ability. Although the *FLICM* and KW*FLICM* algorithms do not need to set additional parameters, their estimation of pixel attenuation in the neighborhood is still inaccurate, and part of the image information is not fully utilized, resulting in unsatisfactory anti-noise performance of the algorithm and inaccurate cutting results.

It is worth mentioning that nature-inspired computing is attracting more and more attention. Metaheuristic algorithms can find the segmentation threshold more accurately in image segmentation [18]. Two of the most popular algorithms are swarm intelligence (SI) and evolutionary algorithms (EAs). The stability and accuracy of the grey wolf optimization (GWO) algorithm has been clearly proved to be better than particle swarm optimization (PSO), gravitational search algorithm (GSA), differential evolution (DE), evolutionary programming (EP) and evolution strategy (ES), which are all well-known meta-heuristics [19]. Using the GWO algorithm to find the initial clustering center of the image is very beneficial. The initial clustering center can be found more accurately and stably to be prepared for subsequent calculations. However, in some cases, due to the lack of diversity of wolves, the GWO algorithm still faces the risk of local extreme stagnation when the traditional GWO algorithm cannot realize the smooth transition from exploration potential to development potential through multiple iterations. In the literature [20], the improved differential evolution grey wolf optimization (DE*GWO*) algorithm is used to find the segmentation threshold of synthetic aperture radar (SAR) images, and good segmentation effect is obtained. The Lévy GWO (LGWO) algorithm [21] is utilized to solve the global problem by introducing Lévy flight algorithm and balancing the exploration and development stage of the algorithm.

In this paper, an adaptive fuzzy c-means segmentation image algorithm based on global spatial information (the A*FCM*_GSI algorithm) is proposed. The LGWO algorithm was adopted to calculate the initial clustering center. By combining the neighborhood and non-neighborhood information of the image, the corresponding weight was calculated adaptively, and neighborhood spatial information was added to the clustering model. The information entropy is utilized to automatically balance the relationship between the pixel information and the non-neighborhood spatial information. The segmentation results of different images show that this algorithm can achieve better segmentation performance under intense noise.

## 2. Related Work and Background

### 2.1. Traditional FCM Algorithm

The fuzzy clustering algorithm (*FCM*) was first proposed by Dunn, then expanded by Bezdek et al. and has been applied in many fields. In essence, the *FCM* algorithm classifies samples according to the intensity of membership, and the objective function is weighted distance sum, which is defined as follows:

(1) $$J_{FCM}=\sum_{i=1}^{c}\sum_{j=1}^{n}u_{ij}^{m}d^{2}\left(x_{j},v_{i}\right)$$

where c is the number of clusters, n is the number of pixels in the image, $u_{ij}$ denotes the membership degree of $x_{j}$ in the $i^{th}$ cluster, has a value inside [0,1] and satisfies the condition $0\leq u_{ij}\leq 1,\sum_{i=1}^{c}u_{ij}=1$, m is the fuzzy weight index and is generally a value of 2, $d\left(x_{j},v_{i}\right)$ represents the Euclidean distance from the $j^{th}$ pixel $x_{j}$ to the $i^{th}$ clustering center $v_{i}$.

While the FCM algorithm is built on the initial parameter set, it determines the minimum objective function $J_{FCM}$ through an iterative process. u and v are described as in Equations (2) and (3):

(2) $$u_{ij}=\frac{1}{\sum_{k=1}^{c}{\left(\frac{d_{ij}}{d_{kj}}\right)}^{\frac{2}{m-1}}}$$

(3) $$v_{i}=\frac{\sum_{j=1}^{n}u_{ij}^{m}x_{j}}{\sum_{j=1}^{n}u_{ij}^{m}}$$

where $u_{ij}$, $v_{i}$ denote the membership function and cluster centers, respectively.

The *FCM* algorithm calculates the membership of each pixel in the image by minimizing the objective function, but the *FCM* algorithm ignores the contribution of neighborhood pixels to the clustering center, so it is sensitive to noise.

### 2.2. FCM_S Algorithm

The *FCM*_S algorithm [12] overcomes the influence of noise on image clustering to a certain extent by introducing neighborhood space constraints. The objective function of *FCM*_S is as follows:

(4) $$J_{FCM\_S}=\sum_{i=1}^{c}\sum_{j=1}^{n}u_{ij}^{m}{\left\|x_{j}-v_{i}\right\|}^{2}+\frac{\alpha }{N_{R}}\sum_{i=1}^{c}\sum_{j=1}^{n}u_{ij}^{m}\sum_{r\in N_{j}}{\left\|x_{r}-v_{i}\right\|}^{2}$$

(5) $$u_{ij}=\frac{{\left(d_{ij}^{2}+\frac{\alpha }{N_{R}}\sum_{r\in N_{j}}d_{ir}^{2}\right)}^{-1/(m-1)}}{\sum_{k=1}^{c}{\left(d_{ij}^{2}+\frac{\alpha }{N_{R}}\sum_{r\in N_{j}}d_{ir}^{2}\right)}^{-1/(m-1)}}$$

(6) $$v_{i}=\frac{\sum_{j=1}^{n}u_{ij}^{m}\left(x_{j}+\frac{\alpha }{N_{R}}\sum_{r\in N_{j}}x_{r}\right)}{\left(1+\alpha \right)\sum_{j=1}^{n}u_{ij}^{m}}$$

where c is the number of clusters, n is the number of pixels in the image, $u_{ij}$ denotes the membership degree of $x_{j}$ in the $i^{th}$ cluster, has a value inside [0,1] and *t* satisfies the condition $0\leq u_{ij}\leq 1,\sum_{i=1}^{c}u_{ij}=1$, m is the fuzzy weight index and is generally a value of 2, $\left\|x_{j}-v_{i}\right\|$ represents the Euclidean distance from the $j^{th}$ pixel $x_{j}$ to the $i^{th}$ clustering center $v_{i}$, $N_{R}$ is the window cardinality, $x_{r}$ denotes the neighborhood pixel set centered on the $j^{th}$ pixel $x_{j}$, α is the influence factor of neighborhood spatial information on the center pixel. The larger the value of α, the greater the role of neighborhood spatial information in the clustering process, and vice versa. When α is 0, the *FCM*_S algorithm reverts to the *FCM* algorithm.

The *FCM*_S algorithm has a certain inhibitory effect on noise, but the algorithm needs to set up the parameters between the noise removal and the preservation of the image details; in general, different parameters are required for different images, and as these parameters are selected by a large number of experiments, the adaptive ability of the algorithm is poor. Because the *FCM*_S algorithm needs to calculate the neighborhood information of the pixels in each iteration, the time complexity of the *FCM*_S algorithm is high. It is still a difficult and hot topic to reduce the computation time of the algorithm under the premise of ensuring segmentation precision.

### 2.3. FLICM Algorithm

The *FLICM* algorithm [15] avoids the introduction of supervised parameters and enhances the practicability of the algorithm when calculating the contribution of neighborhood information to the pixels of the center. The *FLICM* algorithm combines the spatial and grey information about the neighborhood pixels by constructing the fuzzy factor $G_{ki}$, which strengthens the insensitivity of the algorithm to the noise. The expression of $G_{ki}$ is as follows:

(7) $$G_{ki}=\sum_{\begin{array}{l} j\notin N_{i}\\ i\neq j \end{array}}\frac{1}{d_{ij}+1}{\left(1-u_{kj}\right)}^{m}{\left\|x_{j}-v_{k}\right\|}^{2}$$

where $d_{ij}$ is the Euclidean distance between neighborhood pixels $x_{j}$ and center pixel $x_{i}$, $1/\left(d_{ij}+1\right)$ denotes the spatial action intensity of neighborhood pixels on central pixels, $u_{kj}$ is the membership strength of neighborhood pixels $x_{j}$ relative to the $k^{th}$ cluster center $v_{k}$, $\left\|x_{j}-v_{k}\right\|$ denotes the Euclidean distance between neighborhood pixels $x_{j}$ and cluster center $v_{k}$ and m is the fuzzy weight index and is generally a value of 2. The objective function of the *FLICM* algorithm is defined as follows:

(8) $$J_{FLICM}=\sum_{i=1}^{N}\sum_{k=1}^{c}\left[u_{ki}^{m}{\left\|x_{i}-v_{k}\right\|}^{2}+G_{ki}\right]$$

The objective function of the *FLICM* algorithm is different from that of the *FCM* algorithm, but their clustering centers are the same. By transplanting the cluster center of the *FCM* algorithm, the iterative updating of the cluster center is completed. Fuzzy membership and the clustering center of *FLICM* algorithm is as follows:

(9) $$u_{ki}=\frac{1}{\sum_{j=1}^{c}{\left(\frac{{\left\|x_{i}-v_{k}\right\|}^{2}+G_{ki}}{{\left\|x_{i}-v_{j}\right\|}^{2}+G_{ji}}\right)}^{1/\left(m-1\right)}}$$

(10) $$v_{k}=\frac{\sum_{i=1}^{N}u_{ki}^{m}x_{i}}{\sum_{i=1}^{N}u_{ki}^{m}}$$

Although *FLICM* improves the fuzzy factor and makes the algorithm more adaptive, it has the disadvantages of slow convergence speed, more iterations and more sensitive to salt and pepper noise.

### 2.4. Parallel LGWO Algorithm

The LGWO algorithm [21] uses Lévy flight algorithm to help GWO obtain the global optimal solution. It has strong global convergence and robustness and the stagnation problem can also be relieved. By integrating the Lévy flight algorithm into LGWO, the search capabilities are stronger because each pioneer wolf gets the chance to survive and then share its observed info with other hunters during the next steps of the searching process. Using LGWO to search for a set of global optimal centers can significantly explore and localize the possible situations of the victim more effectually. However, the LGWO algorithm is a probabilistic search algorithm, and its performance is affected by control parameters such as the size of the wolves and random mutation probability. As the algorithm requires a large wolf pack size, it needs to continuously calculate the fitness function. In this paper, the computational complexity is related to the number of image clusters, and the computational complexity is $O\left(NP\times T\_LGWO\times C\right)$, where NP is the number of wolves, T_LGWO is the total number of iterations, and C is the number of clustering centers of the image. Therefore, this paper proposes a parallel LGWO algorithm to improve the reliability and efficiency of the algorithm. The computation time of the algorithm is greatly reduced.

**Figure 1:**
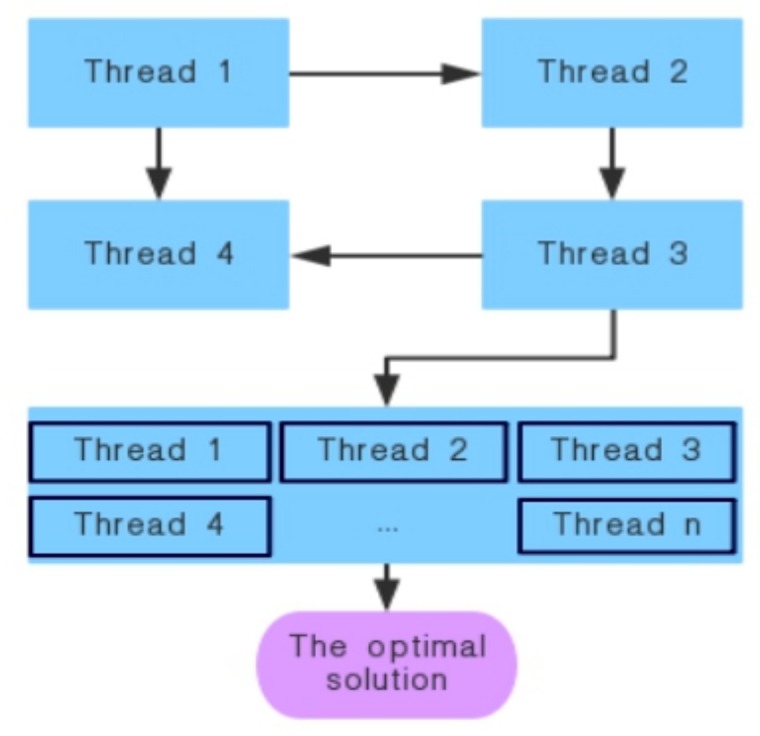
Parallel Lévy grey wolf optimization (LGWO) algorithm block diagram.

In nature, wolves can be thought of as being made up of several subgroups, so groups can be divided into several subgroups. Each subgroup contains multiple individuals, and each subgroup is allocated a processor to execute the search process independently in parallel. The best individuals in each subgroup migrate to neighboring subgroups after a certain period of time, a phenomenon known as “drift”. This is the coarse-grained parallel LGWO algorithm and its block diagram is shown in Figure 1:

In this paper, parallel computing is used to speed up the computation of the program. Parallel LGWO algorithm flow is shown in Figure 1. Each wolf subpopulation is assigned a processing core to perform the search independently, and the optimal individuals are recorded after each iteration. The best individuals in each subpopulation will migrate to the adjacent subpopulation after a certain number of iterations. The optimal individual will be obtained after the completion of the iteration.

## 3. The Proposed Methods

### 3.1. Initial Cluster Center

A parallel LGWO algorithm is used to solve the initial clustering center of the original image. The pseudo-code of the initial image clustering center estimated by the parallel LGWO algorithm is as follows:

Input: Image data

(1) Determine the initial swarm size *NP* and the number of iterations *T_LGWO*. The population is initialized into *NP*_s subpopulations, and the corresponding number of threads is opened up. Each thread is responsible for one subpopulation. Each subpopulation is iterated *L* times to transfer its best individuals to the adjacent subpopulation.
(2) Randomly generate the initial subpopulations of wolves
(3) Initialize temporal parameter *a*, random value p, random vectors *A*, *C*
(4) Compute the fitness of each wolf
(5) Set to be the best wolf
(6) Set to be the second best wolf
(7) while (*t < T_LGWO*) or (stopping condition) do
(8) for each wolf
(9) Update the position of current wolves
(10) perform the greedy selection(GS)
(11) end for
(12) Update parameters *a*, *p*, *A*, *C*
(13) Compute the fitness of each wolf
(14) Update,
(15) The number of iterations *t* = *t* + 1
(16) if modulo operation mod (*t*,*L*) = 0, transfer the best individuals of each subpopulation to adjacent subpopulations.
(17) end while
(18) Walk through the optimal solution in each subpopulation, find a global optimal solution, as the final solution.
(19) Return

Output: Partition matrix, cluster center

### 3.2. Fast Non-Local Mean Denoising

Non-local mean (nl-means) [22] is a useful denoising technique. This method makes full use of the redundant information in the image and can preserve the details of the image to the greatest extent while denoising. The basic idea is that the current pixel estimate is a weighted average of pixels in the image with similar neighborhood structures. nl-means use the non-local spatial information of image pixels to filter the original image, and the formula is as follows:

(11) $$\eta_{j}=\sum_{p\in W_{j}^{r}}w_{jp}x_{p}$$

where $\eta_{j}$ is the pixel of the filtered image, $W_{j}^{r}$ denotes the pixel area with pixel j as the center and window size is r×r, $x_{p}$ is the neighborhood pixel in the window, $w_{jp}$ is the weight determined by non-local spatial information, and its size depends on the similarity between the center pixel block and the neighborhood pixel block, and $0\leq w_{jp}\leq 1,\sum_{p\in W_{j}^{r}}w_{jp}=1$.

The formula of $w_{jp}$ is as follows:

(12) $$w_{jp}=\frac{1}{Z_{j}}\exp\left(\frac{-{\left\|x(N_{j})-x(N_{p})\right\|}_{2,\alpha }^{2}}{h_{j}h_{p}}\right)$$

where $N_{j}$ is the pixel region centered on pixel $x_{j}$, $x\left(N_{j}\right)$ denotes the vector composed of all pixels in the central pixel region, $x\left(N_{p}\right)$ denotes the vector composed of all pixels in the neighborhood pixel region, ${\left\|x\left(N_{j}\right)-x\left(N_{p}\right)\right\|}_{2,\alpha }^{2}$ is the similarity between the center pixel block and the neighborhood pixel block, α is the standard deviation of the gaussian kernel function, reflecting the spatial structure between the center pixel and the neighborhood pixel, and $h_{j}$ and $h_{p}$ are the filtering attenuation parameters of the central pixel region and the adjacent pixel region, respectively, which can be adjusted appropriately according to the noise intensity. The filtering attenuation parameter $h_{j}$ is obtained according to the adaptive grey level difference in the pixel block [23], and the formula is as follows:

(13) $$h_{j}={\left\|x_{j}-x\left(N_{jl}\right)\right\|}_{2,\alpha }$$

where $x_{j}$ is the center pixel of the pixel block $N_{j}$, $x\left(N_{jl}\right)$ is the neighborhood pixel $x_{l}$ of $x_{j}$ in the same pixel block, and $h_{j}$ reflects the similarity between the neighborhood pixel and the center pixel through the grey level difference in the pixel block. $h_{p}$ can also be calculated using the same principle. The greater the difference between the neighborhood pixel and the center pixel in the pixel block, the more serious the noise pollution of the pixel block will be, and the greater the filtering intensity of the pixel block, and vice versa. $Z_{j}$ is the normalized constant, defined as follows:

(14) $$Z_{j}=\sum_{p\in W_{j}^{r}}\exp\left(\frac{-{\left\|x(N_{j})-x(N_{p})\right\|}_{2,\alpha }^{2}}{h_{j}h_{p}}\right)$$

where $h_{j}$ and $h_{p}$ make use of the greyscale statistical information of the central pixel block and the neighboring pixel block, respectively, and adjust the filtering attenuation parameters adaptively. $w_{jp}$ is to determine the similarity between the center pixel and the neighborhood pixel by using the redundant information of the image. The closer the center pixel is to the neighborhood pixel, the greater the weight $w_{jp}$ corresponding to the neighborhood pixel will be, and vice versa. Non-local spatial information can avoid the loss of detail information caused by the larger local neighborhood window, and this method can play a better guiding role in the noisy image.

nl-means has good denoising effect, but the maximum defect of this algorithm is too high in computational complexity. Assuming that the image is a total of M pixels, the size of the search window is R×R, the neighborhood window size is r×r. The complexity of the nl-means algorithm is $O\left(MR^{2}r^{2}\right)$. Therefore, integral image technology is used to accelerate this algorithm [24]. First, an integral image about pixel difference is constructed:

(15) $$S_{t}(x)=\sum_{\left\{\kappa =(\kappa 1,\kappa 2)\in M^{2}:0\leq \kappa 1\leq x1,0\leq \kappa 2\leq x2\right\}}s_{t}\left(x\right),x=\left(x_{1},x_{2}\right)$$

(16) $$s_{t}\left(x\right)={\left\|x(N_{j})-x(N_{j+t})\right\|}_{}^{2}$$

(17) $$\begin{array}{l} {\left\|x(N_{j})-x(N_{p})\right\|}^{2}=\frac{1}{r^{2}}\left(S_{t}(x_{1}+rs,x_{2}+rs)+\right.S_{t}(x_{1}-rs-1,x_{2}-rs-1)\\ -S_{t}(x_{1}+rs,x_{2}-rs-1)-\left.S_{t}(x_{1}-rs-1,x_{2}+rs)\right) \end{array}$$

where the search window side length is R=2∗Rs+1, and the search window side half-length is RS. The neighborhood window side length is r=2∗rs+1,and the neighborhood window side half-length is rs. In order to reduce the space complexity, the above algorithm takes the offset as the outermost loop, and only needs to calculate the integral image in one offset direction at a time, and then process the integral image. After the above processing, the complexity of the whole algorithm will be reduced to $O\left(MR^{2}\right)$.

### 3.3. Improved Value Function

The A*FCM*_GSI algorithm makes full use of the neighborhood and non-neighborhood information about pixels and adaptively adjusts the corresponding weight. The main objective function is as follows:

(18) $$J\left(U,V\right)=\sum_{j=1}^{N}\sum_{k=1}^{c}\left[u_{kj}^{m}(1-\beta_{j})d_{r}^{2}(x_{i},v_{k})+\right.u_{kj}^{m}\beta_{j}d_{r}^{2}(\eta_{j},v_{k})+\left.K_{kj}\right]$$

where c is the number of clusters, N is the number of pixels in the image, $u_{kj}$ denotes the membership degree of $x_{j}$ in the $i^{th}$ cluster and has a value inside [0,1], m is the weighting exponent on each fuzzy membership and generally has a value of 2,$v_{k}$ is the $i^{th}$ cluster center, $\eta_{j}$ is the pixel of the image after fast non-local mean processing and filtering, $\beta_{j}$ is the adjustment parameter calculated by information entropy, and $d_{r}^{2}\left(x_{i},v_{k}\right)$ is the improved distance measure. Using the Lagrange multiplier method to minimize the value function, the fuzzy membership degree $u_{kj}$ and clustering center $v_{k}$ can be obtained as:

(19) $$u_{kj}=\frac{1}{\sum_{k=1}^{c}{\left(\frac{(1-\beta_{j})(1-r(x,v_{k}))+\beta_{j}(1-r(\eta ,v_{k}))+K_{ki}}{(1-\beta_{j})(1-r(x,v_{k}))+\beta_{j}(1-r(\eta ,v_{k}))+K_{ki}}\right)}^{1/\left(m-1\right)}}$$

(20) $$v_{k}=\frac{\sum_{j=1}^{N}\left(u_{kj}^{m}r(x_{j},v_{k})(1-\beta_{j})x+u_{kj}^{m}r(\eta_{j},v_{k})\beta_{j}\eta \right)}{\sum_{j=1}^{N}\left(u_{kj}^{m}r(x_{j},v_{k})(1-\beta_{j})+u_{kj}^{m}r(\eta_{j},v_{k})\beta_{j}\right)}$$

Traditional Euclidean distance cannot solve the problem of noise sensitivity of the algorithm [25]. Although nuclear induced distance [26] can make up for the deficiency of Euclidean distance to some extent, it is sometimes difficult to overcome the influence of noise on clustering performance and it cannot fundamentally solve the problem of noise sensitivity. In order to make up for this deficiency, an improved distance measurement method is adopted in this paper, specifically as follows:

(21) $$d_{r}(x_{i},v_{k})=\sqrt{1-r(x_{i},v_{k})}$$

(22) $$r(x_{i},v_{k})=\exp(-\psi {\left\|x_{i}-v_{k}\right\|}^{2})$$

(23) $$\psi ={\left(\frac{\sum_{i=1}^{n}{\left\|x_{i}-\bar{x}\right\|}^{2}}{n}\right)}^{-1}$$

(24) $$\bar{x}=\frac{1}{n}\sum_{i=1}^{n}x_{i}$$

The improved distance measurement method is based on robust statistics theory and has strong stability to noise or outliers. Although the distance measurement is similar to the nuclear induced distance in form, its essence is still processed in the original image space, and the pixels are not mapped to the high-dimensional feature space [27].

Parameter $\beta_{j}$ can adjust the balance between pixel information and non-neighborhood spatial information. The calculation method of this parameter is as follows:

(25) $$\beta_{j}=\frac{E_{j}-E_{\min}}{E_{\max}-E_{\min}},$$

(26) $$E_{j}=-\sum_{k}^{c}u_{kj}\log_{2}u_{kj},$$

where $E_{j}$ represents the information entropy of the $j^{th}$ pixel, and $E_{\max}$ and $E_{\min}$ respectively represent the maximum and minimum information entropy of all pixel points. Equation (25) can map the range of information entropy to [0,1]. If the $j^{th}$ point belongs to a certain class explicitly, the entropy corresponding to that point is relatively small. If the membership degree of this point is average, indicating that it does not clearly belong to a certain class, the corresponding entropy of this point is relatively large, which can increase the weight of non-neighborhood pixel information.

In the literature [15], the fuzzy factor uses the spatial distance between the neighborhood pixel and the center pixel to measure the degree of influence of the neighborhood pixel. The spatial distance is defined as follows:

(27) $$\delta_{sd}=\frac{1}{d_{ij}+1}$$

where $\delta_{sd}$ denotes the spatial intensity of neighborhood pixels on central pixels. However, spatial distance alone cannot accurately measure the influence of neighborhood points on the center points. By introducing the local variation coefficient that has an important influence on the central pixel, the variation coefficient of the local window is defined as:

(28) $$\delta_{sv}=1-\log_{2}(\sqrt{\varphi_{j}}+1)$$

(29) $$\varphi_{j}=\frac{C_{j}-C_{\min}}{C_{\max}-C_{\min}}$$

(30) $$C_{u}=\frac{V(x)}{{\left(\bar{x}\right)}^{2}}$$

where V(x) is the variance of grey value in a local window, $\bar{x}$ denotes the average grey level of neighborhood pixels, $C_{\min}$ is the minimum coefficient of variation in all local windows of an image, $C_{\max}$ is the maximum value, $\delta_{sv}$ denotes the discretization of pixel grey values in the local window of neighborhood points and has a value inside [0,1], $\delta_{sv}$ is inversely proportional to $\varphi_{j}$ when the value of $\varphi_{j}$ is close to 0, the $\delta_{sv}$ value is close to 1, and the logarithmic function can ensure that when the $\varphi_{j}$ is far away from 0, $\delta_{sv}$ decreases rapidly; when $\varphi_{j}$ is close to 1, $\delta_{sv}$ is close to 0. That is to say, when the neighborhood point is seriously affected by the noise or at the edge, the value of the $\delta_{sv}$ is close to 0 and the influence of the neighborhood point on the center point is also close to 0, and the value of $\delta_{sv}$ is larger when neighborhood points of the window are smooth, the influence of the neighborhood point on the center point is larger.

Based on testing and analysis, the influence of neighborhood pixels on the center point is redefined as follows:

(31) $$\delta_{ij}=\frac{\delta_{sd}{}^{2}+\delta_{sv}{}^{2}}{\delta_{sd}+\delta_{sv}}$$

According to Equation (31), the influence of a pixel’s neighborhood spatial information on image segmentation is defined as:

(32) $$K_{ki}={\sum_{j\in N_{i}}\delta_{ij}(1-u_{kj})}^{m}{\left\|x_{j}-v_{k}\right\|}^{2}$$

The specific steps of the A*FCM*_GSI algorithm are as follows:

Step 1: Determine the number of clusters c, fuzzy weighted index m, the number of iterations *T*_max, the iterative termination threshold ε, the size of the search window R∗R, the size of the neighborhood window r∗r, and the number of current iterations *t* = 1;
Step 2: The initial clustering center $V^{\left(0\right)}$ is obtained by the LGWO algorithm, calculate the filtered image $\eta_{j}$.
Step 3: Initialization of the membership degree matrix $U^{\left(0\right)}$.
Step 4: Compute weight parameter $\beta_{j}$.
Step 5: Compute the new objective function value J.
Step 6: Update membership degree matrix U by Equation (19).
Step 7: Update cluster centers V by Equation (20).
Step 8: If $\left\|J^{\left(t+1\right)}-J^{\left(t\right)}\right\|<\varepsilon$ or the current iteration number t>T_max, then terminate the iteration, output the membership matrix U and the cluster center V; otherwise, return Step 4 and continue the next iteration.

## 4. Experimental Results and Performance Analysis

### 4.1. Evaluation Index of Fuzzy Clustering Algorithm

In order to verify the effectiveness of the clustering algorithm, scholars have proposed a variety of evaluation indicators [28,29,30,31,32,33]. *SA* (segmentation accuracy) and *CS* (comparison score) are widely used and approved.

(33) $$SA=\frac{G\cap S}{S}$$

(34) $$CS=\frac{G\cap S}{G\cup S}$$

where SA represents the proportion of pixels in the region detected by the segmentation algorithm in the whole region and CS is a measure of similarity. The area of the given annotation is represented by G. The pixel area detected by the algorithm is represented by S. As the natural image has no standard segmentation results, the corresponding segmentation accuracy and comparison scores cannot be calculated. In order to effectively evaluate the segmentation results of natural images, the *PSNR* (peak signal to noise ratio) and *MSSIM* (mean structural similarity) are introduced in this paper.

(35) $$PSNR=10\log_{10}(\frac{{\left(2^{n}-1\right)}^{2}}{MSE})$$

(36) $$MSE=\frac{1}{H\times W}\sum_{i=1}^{H}\sum_{j=1}^{W}{\left(X(i,j)-Y(i,j)\right)}^{2}$$

(37) $$MSSIM=\frac{1}{N}\sum_{k=1}^{N}SSIM(x_{k},y_{k})$$

(38) $$SSIM(X,Y)=\frac{\left(2\mu_{X}\cdot \mu_{Y}+C_{1})(2\sigma_{XY}+C_{2}\right)}{\left(\mu_{X}^{2}+\mu_{Y}^{2}+C_{1})(\sigma_{X}^{2}+\sigma_{Y}^{2}+C_{2}\right)}$$

(39) $$\mu_{X}=\sum_{i=1}^{H}\sum_{j=1}^{W}\omega_{ij}X(i,j)$$

(40) $$\sigma_{X}={\left(\sum_{i=1}^{H}\sum_{j=1}^{W}\omega_{ij}(X(i,j)-\mu_{X})\right)}^{1/2}$$

(41) $$\sigma_{XY}=\sum_{i=1}^{H}\sum_{j=1}^{W}\omega_{ij}(X(i,j)-\mu_{X})(Y(i,j)-\mu_{Y})$$

where MSE denotes the mean square error of the current image X and the reference image Y, and H and W are the height and width of the image respectively. The unit of *PSNR* is dB; the larger the value, the smaller the distortion. N is equal to the number of bits per pixel, and the average grey level image is 8; that is, the grey scale of pixels is 256. $\omega_{ij}$ is the weight of each window, H and W are the height and width of the image respectively, $\mu_{X}$ and $\mu_{Y}$ are the mean values of images X and Y respectively. $\sigma_{X}$ and $\sigma_{Y}$ denote the variance of X and Y respectively, and $\sigma_{XY}$ indicates the covariance of image X and Y. $C_{1}$,$C_{2}$ and $C_{3}$ are constants; in order to avoid the denominator being 0, they are usually defined as $C_{1}={\left(K_{1}*L\right)}^{2}$,$C_{2}={\left(K_{2}*L\right)}^{2}$, $C_{3}=C_{2}/2$, and $K_{1}=0.01$, $K_{2}=0.03$, L=255. In practical applications, the image can be partitioned by a sliding window. The total number of blocks is N. Considering the influence of window shape to the partition, the mean, variance and covariance of each window are calculated by weighting. The Gauss kernel is usually used, the structure similarity of the corresponding block is computed, and the structure similarity (*SSIM*) of the corresponding block is calculated. Finally, the average value (*MSSIM*) is used to measure the structural similarity of two images.

*PSNR* is the most widely used image objective evaluation index, but it is based on the error between the corresponding pixels, which is based on the error of sensitive image quality evaluation. Because the human eye is more sensitive to the contrast difference of the spatial frequency, the sensitivity of the human eye to the contrast difference is higher than that of the color, but the perception of the human eye is affected by the surrounding area in a region. Therefore, it often appears that the evaluation results are not consistent with the subjective feelings of the people. *MSSIM* is used to measure similarity between two images. One of the two images used by *SSIM* is an unimpressed undistorted image and the other is a distorted image. It is another excellent algorithm for image quality evaluation.

### 4.2. Algorithm Performance Test

In order to verify the effectiveness of the algorithm, synthetic images, optical images, and remote sensing images were used to conduct experiments, respectively. Images polluted by synthetic noise (composed of salt and pepper noise with density = 0.02, speckle noise with variance = 0.005, and Gaussian noise with mean = 0 and variance = 0.01). This algorithm is compared with several algorithms such as *FCM*_S [12], FG*FCM* [14], *FLICM* [15], and KW*FLICM* [17] to test the segmentation effect of the algorithm. The parameters in numerous comparison algorithms are set according to corresponding documents. In order to achieve good experimental results, the relevant parameters in this experiment are set as follows: m=2, $\varepsilon =10^{-5}$, T_max=300, search window size R=5 and neighborhood window size r=3. Among them, the iteration termination threshold ε is a smaller number, and its value is usually selected based on human experience. The results obtained from experiments are the mean value of the algorithm running several times.

**Figure 2:**
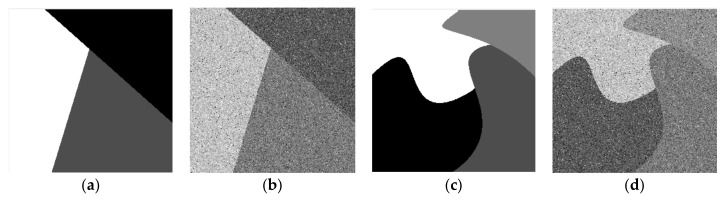
(**a**) Noise-free synthetic image 1; (**b**) synthetic image 1 polluted by synthetic noise; (**c**) noise-free synthetic image 2; (**d**) synthetic image 2 polluted by synthetic noise.

In the segmentation and comparison experiment of synthetic image 1 and 2, clustering numbers of all algorithms are set as 3 and 4, respectively. These synthetic images and their noise-polluted images are shown in Figure 2.

**Figure 3:**
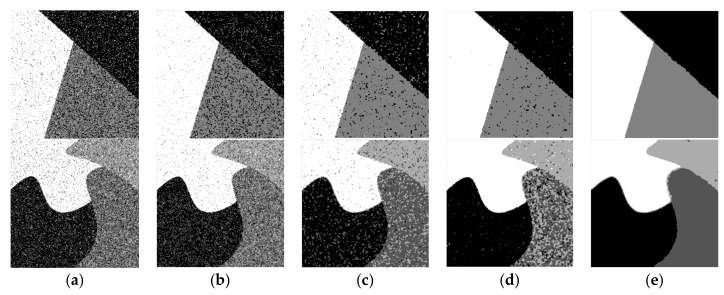
(**a**) segmented image by *FCM*_S; (**b**) segmented image by FG*FCM*; (**c**) segmented image by *FLICM*; (**d**) segmented image by KW*FLICM*; (**e**) segmented image by A*FCM*_GSI.

**Figure 4:**
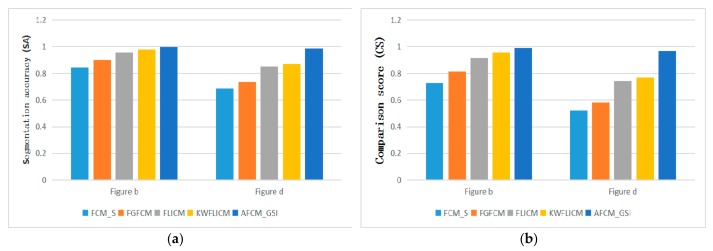
SA and CS of five algorithms on noisy synthetic image 1 and 2.

In the comparison experiment of synthetic images, Figure 3 shows the segmentation effect of several different segmentation methods. The SA and CS of different methods can be more intuitively compared through Table 1 and Figure 4. Moreover, the more complex the image is, the lower the segmentation accuracy will be. The values of SA and CS in the proposed method are the largest and the segmentation effect is the best. By combining the neighborhood and non-neighborhood information of pixels, the relationship between noise suppression and edge preservation can be well balanced. The segmentation result is very similar to the original image and is superior to other algorithms in visual quality and segmentation performance. Among several algorithms, the *FCM*_S algorithm has the weakest noise reduction ability. Although the *FCM*_S algorithm introduces the neighborhood spatial information, the processed image has too much noise. The segmentation performance is not high enough under the noise condition, and the segmentation result is not ideal. The segmentation effect of the FG*FCM* algorithm is better than that of the *FCM*_S algorithm, but there are still more noise points in the image and the edges are more fuzzy, so the ideal segmentation effect and anti-noise performance cannot be obtained. The *FLICM* algorithm and the KW*FLICM* algorithm better consider the neighborhood information of the pixel, with higher segmentation quality and better visual effect. To sum up, the algorithm proposed in this paper can achieve a better segmentation effect and anti-noise ability.

**Figure 5:**
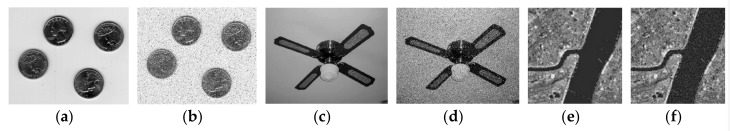
(**a**) Noise-free natural image 1; (**b**) natural image 1 polluted by synthetic noise; (**c**) noise-free natural image 2; (**d**) natural image 2 polluted by synthetic noise; (**e**) noise-free remote sensing image 1; (**f**) remote sensing image 1 polluted by synthetic noise.

In the segmentation and comparison experiment of natural images and a remote sensing image 1, in order to achieve a better segmentation effect, the clustering number of all algorithms is set to 2. In the segmentation and comparison experiment of natural image 2 and remote sensing image 1, the clustering number of all algorithms is set to 3. These images are public images on the Internet. These images and their noise-polluted images are shown in Figure 5.

**Figure 6:**
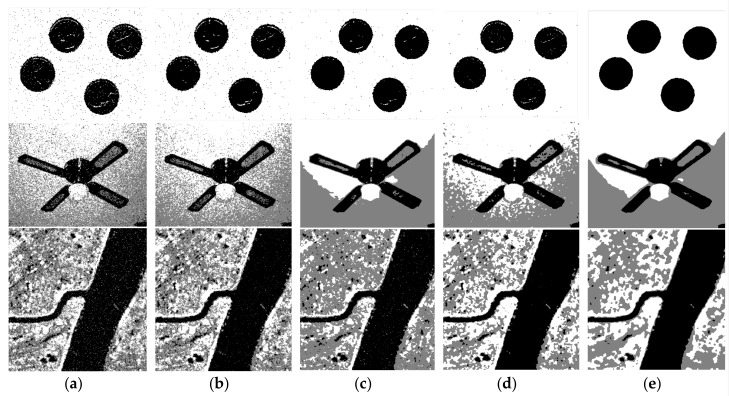
(**a**) segmented image by *FCM*_S; (**b**) segmented image by FG*FCM*; (**c**) segmented image by *FLICM*; (**d**) segmented image by KW*FLICM*; (**e**) segmented image by A*FCM*_GSI.

**Figure 7:**
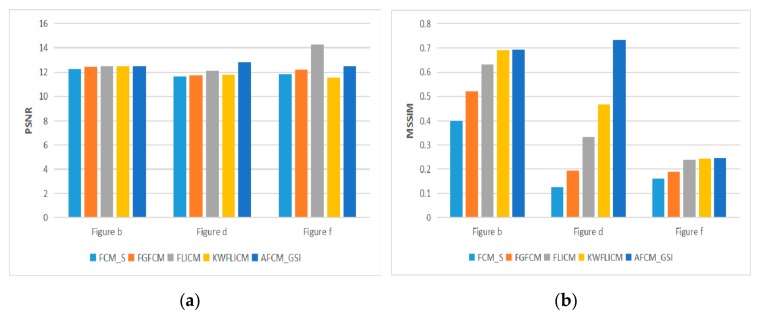
*PSNR* and *MSSIM* of five algorithms on noisy natural images and remote sensing image 1.

**Figure 8:**
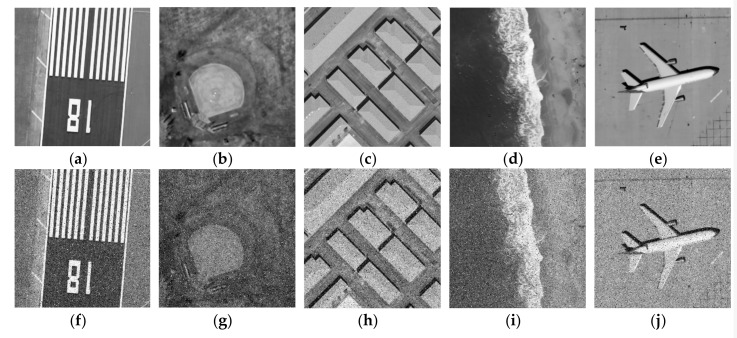
(**a**) Noise-free remote sensing image 2; (**b**) noise-free remote sensing image 3; (**c**) noise-free remote sensing image 4; (**d**) noise-free remote sensing image 5; (**e**) noise-free remote sensing image 6; (**f**) remote sensing image 2 polluted by synthetic noise; (**g**) remote sensing image 3 polluted by synthetic noise; (**h**) remote sensing image 4 polluted by synthetic noise; (**i**) remote sensing image 5 polluted by synthetic noise; (**j**) remote sensing image 6 polluted by synthetic noise;.

**Figure 9:**
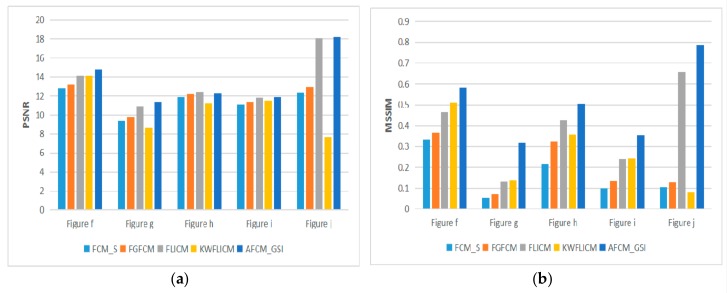
*PSNR* and *MSSIM* of five algorithms on noisy remote sensing images 2–6.

In the segmentation and contrast experiment of different kinds of remote sensing images 2–6, the clustering number of all algorithms is set as 3. These images were manually extracted from large images from the United States Geological Survey (USGS) National Map Urban Area Imagery collection for various urban areas around the country [34]. These images and their synthetic noise-polluted images are shown as follows:

In the comparison experiment of optical images and remote sensing images, the segmentation effect of different algorithms can be observed in Figure 6. PSNR and MSSIM of different algorithms can be compared more clearly and intuitively through Table 2 and Figure 7. In order to verify the availability of the proposed algorithm, segmentation experiments are carried out for different types of remote sensing images in Figure 8. Experimental results in Table 3 and Figure 9 demonstrate the advantages of the proposed algorithm. By comparing the segmentation results of optical images and remote sensing images, the segmentation method proposed in this paper achieves very good segmentation results. Due to the need to balance the denoising performance and image details of the algorithm, the *PSNR* value of the A*FCM*_GSI algorithm is sometimes not the highest among all algorithms, but the *MSSIM* value is the highest among all algorithms. Since *PSNR* does not consider the visual characteristics of human eyes, the similarity measure (*MSSIM*) between two images can represent the quality of image segmentation. Experimental results show that the proposed algorithm has strong denoising ability and the segmented image is very similar to the original image. The algorithm has the highest *MSSIM* value and the best visual effect of image segmentation.

## 5. Conclusions

In this paper, an adaptive image segmentation algorithm based on global spatial information is proposed to improve the anti-noise and precision of image segmentation. By introducing neighborhood and non-neighborhood information of pixels, this method adaptively adjusts the corresponding weight and has good denoising performance. This method uses the LGWO algorithm to roughly cluster the image and get the initial clustering center and utilizes a fast non-local mean algorithm to filter the original image. The adaptive weight assignment strategy is adopted to assign a corresponding weight to each pixel in the neighborhood window and make full use of the local information of the image. The information entropy is used to balance the relationship between the pixel and the non-neighborhood information, and the neighborhood and non-neighborhood information around the pixel is added to the target function as spatial information. The improved distance measure is also used to replace the traditional Euclidean distance. Experimental results show that the above improvements can make the segmentation results more accurate. This paper proves the feasibility of this algorithm, which has the advantages of high segmentation accuracy and good denoising effect.

## Figures and Tables

**Table 1:** Segmentation accuracy (SA) and comparison scores (CS) of five algorithms on noisy synthetic image 1 and 2.

Image	Algorithms	*FCM*_S	FG*FCM*	*FLICM*	KW*FLICM*	A*FCM*_GSI
b	SA	0.8423	0.8976	0.9565	0.9780	**0.9953**
b	CS	0.7275	0.8142	0.9161	0.9569	**0.9907**
d	SA	0.6860	0.7343	0.8508	0.8701	**0.9843**
d	CS	0.5221	0.5802	0.7409	0.7703	**0.9671**

**Table 2:** Peak signal to noise ratio (*PSNR*) and mean structural similarity (*MSSIM*) of five algorithms on noisy natural images and remote sensing image 1.

Image	Algorithms	*FCM*_S	FG*FCM*	*FLICM*	KW*FLICM*	A*FCM*_GSI
b	*PSNR*	12.2791	12.4592	12.5016	12.5148	**12.5284**
b	*MSSIM*	0.3987	0.5205	0.6324	0.6921	**0.6934**
d	*PSNR*	11.6559	11.7341	12.1253	11.7898	**12.7938**
d	*MSSIM*	0.1243	0.1931	0.3340	0.4668	**0.7322**
f	*PSNR*	11.8354	12.2012	**14.2452**	11.5220	12.5273
f	*MSSIM*	0.1608	0.1877	0.2398	0.2450	**0.2471**

**Table 3:** *PSNR* and *MSSIM* of five algorithms on noisy remote sensing images 2–6.

Image	Algorithms	*FCM*_S	FG*FCM*	*FLICM*	KWF*LICM*	A*FCM*_GSI
f	*PSNR*	12.7873	13.2223	14.1081	14.1206	**14.8136**
f	*MSSIM*	0.3332	0.3666	0.4667	0.5086	**0.5829**
g	*PSNR*	9.3817	9.8205	10.8840	8.6311	**11.3334**
g	*MSSIM*	0.0529	0.0721	0.1321	0.1381	**0.3190**
h	*PSNR*	11.8431	12.2115	**12.4235**	11.2166	12.2928
h	*MSSIM*	0.2176	0.3247	0.4278	0.3557	**0.5046**
i	*PSNR*	11.0907	11.3853	11.7925	11.4815	**11.8863**
i	*MSSIM*	0.1009	0.1341	0.2391	0.2414	**0.3552**
j	*PSNR*	12.3548	12.8767	18.0852	7.7038	**18.2051**
j	*MSSIM*	0.1046	0.1306	0.6565	0.0806	**0.7849**
